# Computational Immunogenetic Analysis of Botulinum Toxin A Immunogenicity and HLA Gene Haplotypes: New Insights

**DOI:** 10.3390/toxins17040182

**Published:** 2025-04-06

**Authors:** Eqram Rahman, Parinitha Rao, Munim Ahmed, William Richard Webb, Jean D. A. Carruthers

**Affiliations:** 1 Research and Innovation Hub, Innovation Aesthetics, London WC2H 9JQ, UK; w.r.webb1@icloud.com; 2 The Skin Address, Bengaluru 560080, India; 3 Department of Haematology, Bangabandhu Sheikh Mujib Medical University, Dhaka 1000, Bangladesh; 4 Department of Ophthalmology, University of British Columbia, Vancouver, BC V6T 1Z4, Canada

**Keywords:** botulinum toxin A, immunogenicity, HLA polymorphism, computational immunogenetics, neurotoxin-associated proteins, molecular docking, epitope mapping

## Abstract

Botulinum toxin A (BoNT-A) is widely used in both therapeutic and aesthetic settings; however, the formation of neutralizing antibodies (NAbs) remains a critical concern, leading to treatment failure. Immunogenic responses are known to vary between individuals due to HLA polymorphisms. Although some claim that neurotoxin-associated proteins (NAPs) shield BoNT-A from immune detection or are themselves immunogenic, there is limited molecular evidence supporting either view. This study applies computational immunogenetics to explore BoNT-A immunogenicity, focusing on HLA binding and the influence of accessory proteins. Epitope mapping, molecular docking, and HLA binding predictions were used to evaluate interactions between BoNT-A epitopes and selected class II HLA alleles (HLA-DQA1*01:02, HLA-DQA1*03:03, HLA-DQB1*06:04, HLA-DQB1*03:01, and HLA-DRB1*15:01). To assess the potential immunomodulatory role of NAPs, molecular dynamics (MD) simulations, solvent-accessible surface area (SASA) analysis, and electrostatic potential mapping were also conducted. Key epitopes—L11, N25, and C10—showed strong binding affinities to HLA-DQA1*01:02, HLA-DQB1*06:04, and HLA-DQA1*03:03, indicating a potential immunodominant role. NAPs did not obstruct these epitopes but slightly increased their exposure and appeared to stabilize the toxin structure. Electrostatic mapping and binding free energy calculations suggested no significant immunogenic shift in the presence of NAPs. BoNT-A immunogenicity appears to be influenced by HLA allele variability, reinforcing the value of patient-specific genetic profiling. The presumed immunogenic role of NAPs remains unsubstantiated at the molecular level, underscoring the need for evidence-based evaluation over commercial rhetoric. While these findings provide valuable molecular insight, it is important to acknowledge that they are derived entirely from in silico analyses. As such, experimental validation remains essential to confirm the immunological relevance of these predicted interactions. Nonetheless, this computational framework offers a rational basis for guiding future clinical research and the development of HLA-informed BoNT-A therapies.

## 1. Introduction

Botulinum toxin type A (BoNT-A) is widely recognized for its clinical applications in neurology, pain management, and aesthetic medicine [1]. Despite its efficacy, a significant challenge in long-term BoNT-A therapy is the development of neutralizing antibodies (NAbs), which can lead to treatment resistance and reduced therapeutic outcomes [2,3]. Immunogenicity in biologics, including BoNT-A, is influenced by human leukocyte antigen (HLA) molecules, which present antigenic peptides to T cells, initiating an immune response that can lead to the production of anti-drug antibodies (ADAs) [4]. The degree of immune recognition varies among individuals due to genetic diversity in HLA alleles [5], making it imperative to understand these interactions to mitigate immunogenicity risks in BoNT-A therapy.

Although much has been claimed regarding the immunogenicity of different BoNT-A formulations, there remains a considerable lack of comprehensive and conclusive research [6,7,8,9,10,11,12,13,14,15,16,17,18]. Many assertions rely on speculative claims and counterclaims, often asserting the superiority of one formulation over another without substantial molecular or clinical validation in the absence of robust scientific data [19,20,21,22]. The absence of standardized methodologies in assessing BoNT-A immunogenicity has resulted in inconsistent findings, making it difficult to ascertain the true extent of immunogenic responses in different formulations. This gap in knowledge necessitates an evidence-based approach that moves beyond commercial biases and focuses on objective scientific analysis.

Computational immunogenetics provides a helpful approach for investigating the immunogenic potential of biologic proteins by integrating bioinformatics, structural modeling, and immune prediction tools. In silico techniques like epitope mapping, molecular docking, and HLA binding prediction enable identification of peptide regions likely to be presented by MHC molecules and recognized by T cells. These tools also allow for the visualization of antigen–receptor interfaces and analysis of structural features such as solvent-accessible surface area (SASA), both of which are essential for understanding immune recognition [23,24,25]. These approaches have been successfully applied in the development of peptide-based vaccines, such as for SARS-CoV-2, and in the assessment of immunogenicity in therapeutic monoclonal antibodies and enzyme replacement therapies [26,27,28].

In the context of BoNT-A, computational analyses facilitate the prediction of epitopes with strong binding affinities to HLA class II alleles, helping to identify immunodominant regions that may contribute to variable patient responses. These tools make it possible to assess T-cell epitope recognition and allele-specific HLA presentation—key determinants of ADA formation and immune-mediated treatment failure. The ability to forecast immunogenicity at the individual level has significant implications for personalized medicine, enabling the design of BoNT-A formulations tailored to minimize immunogenic risk and enhance therapeutic safety [29,30,31,32].

There has been an increasing suggestion of HLA-DQA1*01:02 and HLA-DQB1*06:04 in BoNT-A immunogenicity, indicating a potential role in antigen presentation and immune activation. The involvement of these specific alleles in biologic immunogenicity has been observed in various therapeutic contexts, suggesting a need for further investigation [33]. These alleles have been linked to enhanced immune recognition of specific protein structures, due to subtle conformational changes in the protein structure, raising questions about whether certain BoNT-A epitopes are more readily presented to the immune system, contributing to NAb formation. Understanding these genetic associations could provide valuable insights into why certain individuals develop stronger immune responses to BoNT-A than others and whether specific regions of the toxin, particularly those involved in receptor binding and enzymatic function, serve as immunogenic hotspots.

Additionally, the role of specific BoNT-A epitopes in triggering immune recognition remains an area of ongoing inquiry and remains under-researched. While the structure of BoNT-A is highly conserved, subtle variations in epitope accessibility may influence how efficiently HLA molecules, based on allele variations, present antigenic regions to immune cells. It has been proposed that some epitopes located within the heavy chain could exhibit higher immunogenic potential due to their exposure during toxin trafficking [34]. Meanwhile, epitopes in the light chain, particularly those involved in enzymatic cleavage, may also elicit immune responses depending on how they interact with antigen-presenting cells [35]. Identifying which epitopes contribute most significantly to ADA formation is a critical aspect of understanding BoNT-A immunogenicity.

Considerable debate exists regarding the role of non-toxic neurotoxin-associated proteins (NAPs), i.e., accessory proteins, in modulating BoNT-A immunogenicity. While some argue that these proteins enhance the activity of BoNT-A [36,37,38], certain reports suggest that NAPs could contribute to antibody formation, potentially triggering immune responses rather than mitigating them [21,39]. The precise role of these proteins remains controversial, and despite claims of their protective function, no definitive molecular mechanism has been provided to explain how they might shield BoNT-A from immune detection. The ongoing debate underscores the need for empirical investigations that rely on molecular and structural assessments, rather than assumption-based conclusions regarding the impact of accessory proteins.

Although computational immunogenetics has been critiqued for its lack of experimental follow-through, it remains a powerful approach for hypothesis generation and mechanistic insight. By addressing unanswered molecular questions, such in silico frameworks can effectively guide and prioritize future experimental validation. This study aims to provide a rigorous and objective in silico analysis of BoNT-A immunogenicity, leveraging computational immunogenetics to identify and validate immunogenic epitopes. Through a detailed investigation of HLA–epitope interactions, this study seeks to clarify the mechanisms underpinning BoNT-A immune recognition and contribute to a more evidence-driven approach to biologic formulation and administration.

## 2. Results

### 2.1. Epitope–HLA Binding Affinity and Docking Analysis

**Figure 1:**
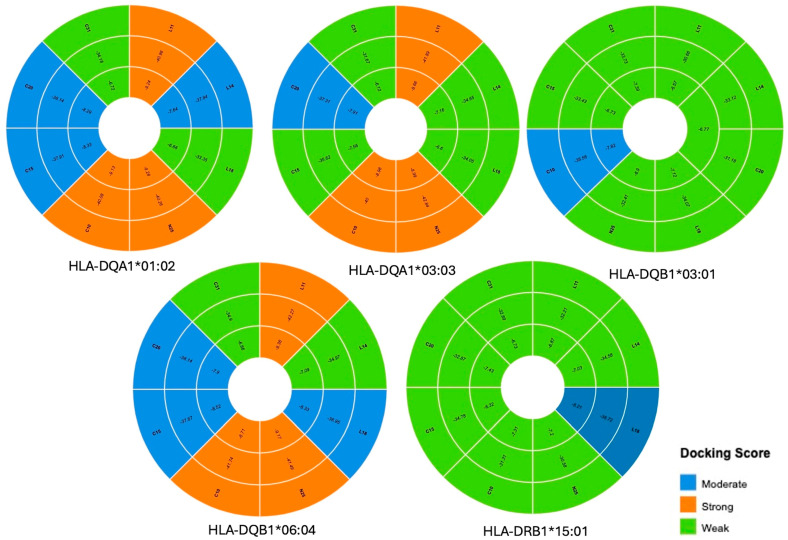
Sunburst chart—epitope–HLA molecular docking, binding affinity, and classification. This sunburst chart shows the molecular docking results between eight BoNT-A epitopes (L11, L14, L18, N25, C10, C15, C20, C31) and five selected HLA class II alleles: *H*LA-DQA1*01:02, HLA-DQA1*03:03, HLA-DQB1*03:01, HLA-DQB1*06:04, and HLA-DRB1*15:01. Each plot represents a specific HLA allele, with individual wedges corresponding to epitopes. Colors indicate the docking score classification—orange (strong binding), blue (moderate binding), and green (weak binding)—based on predicted binding affinities and interaction energies. Strong binders such as L11, C10, and N25 consistently interacted with DQA1*01:02 and DQB1*06:04. In contrast, DQB1*03:01 and DRB1*15:01 mostly exhibited weak binding across epitopes. These results highlight allele-specific differences in epitope recognition, which may contribute to variability in immunogenic responses to BoNT-A.

**Figure 2:**
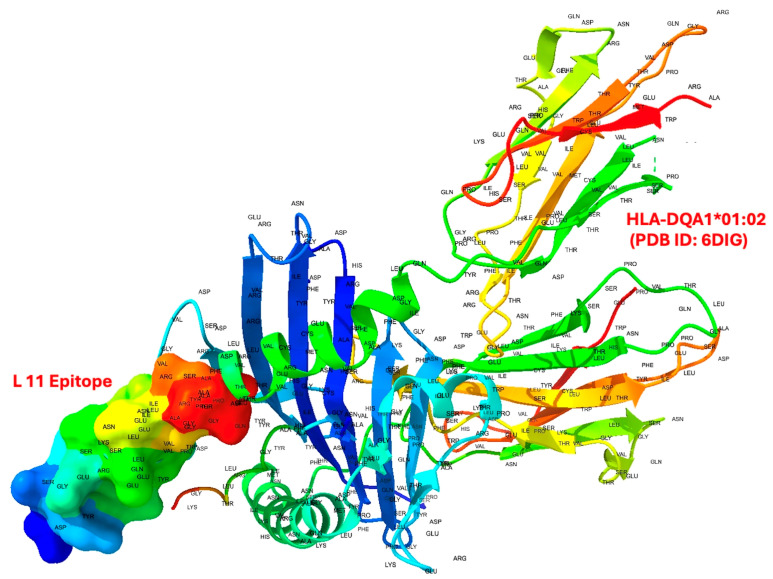
Molecular docking of epitope L11 with HLA-DQA1*01:02 (PDB ID: 6DIG). The image shows the docked complex of the BoNT-A-derived epitope L11 (rendered as a surface model, colored by an electrostatic potential gradient) bound to the HLA-DQA1*01:02 molecule, visualized in ribbon format using UCSF Chimera. The docking was performed following geometry optimization, flexible ligand fitting, and structural alignment. The interaction interface highlights the positioning of the epitope along the HLA–peptide-binding groove, suggesting favorable spatial compatibility for class II antigen presentation. Residue labels are displayed to illustrate the epitope conformation and orientation within the binding cleft.

Molecular docking simulations provided important insights into the binding affinities of BoNT-A epitopes with selected HLA class II proteins from allele variations, including HLA-DQA1*01:02, HLA-DQB1*06:04, HLA-DRB1*15:01, HLA-DQB1*03:01, and HLA-DQA1*03:03. Among the eight examined epitopes, L11, N25, and C10 exhibited the strongest binding affinities across multiple HLA molecules from allele variations, indicating their potential immunodominance. These epitopes showed binding free energies ranging from −6.12 to −9.38 kcal/mol, reinforcing their likelihood of forming stable peptide–HLA complexes. Notably, L11 and N25 had particularly strong interactions with HLA-DQA1*01:02, HLA-DQB1*06:04, and HLA-DQA1*03:03 (Table 1; Figure 1). An example of the molecular docking between L11 and HLA-DQA1*01:02 using UCSF Chimera is also presented in Figure 2.

The docking results revealed significant variability in how BoNT-A epitopes interact with different HLA class II alleles. Notably, epitopes L11, N25, and C10 consistently demonstrated the strongest binding affinities, suggesting their potential immunodominance. These high-affinity interactions are crucial because strong peptide–HLA binding is a known prerequisite for T-cell activation and subsequent ADA development. The interaction energy data further support the stability of these complexes. Conversely, weaker interactions observed with other epitopes (e.g., C31) highlight that not all peptide regions contribute equally to immunogenicity, underscoring the selective nature of HLA-mediated antigen presentation.

### 2.2. Impact of NAPs on Epitope Recognition and Immunogenicity

#### 2.2.1. Structural Interaction of NAPs with BoNT-A Epitopes

Molecular docking simulations revealed differential interactions between individual NAPs and BoNT-A antigenic sites. HA-33, HA-17, and NTNH exhibited low-energy docking scores (−6.8 to −7.4 kcal/mol) near BoNT-A epitopes, indicating moderate affinity but no significant obstruction of antigenic sites.

HA-33 displayed a tendency to bind near the light chain of BoNT-A, particularly around epitopes L11 and L14, but did not completely shield these sites.

HA-17 and HA-70 associated with the heavy chain, forming transient interactions with epitopes N25 and C10, but did not reduce SASA significantly.

**Figure 3:**
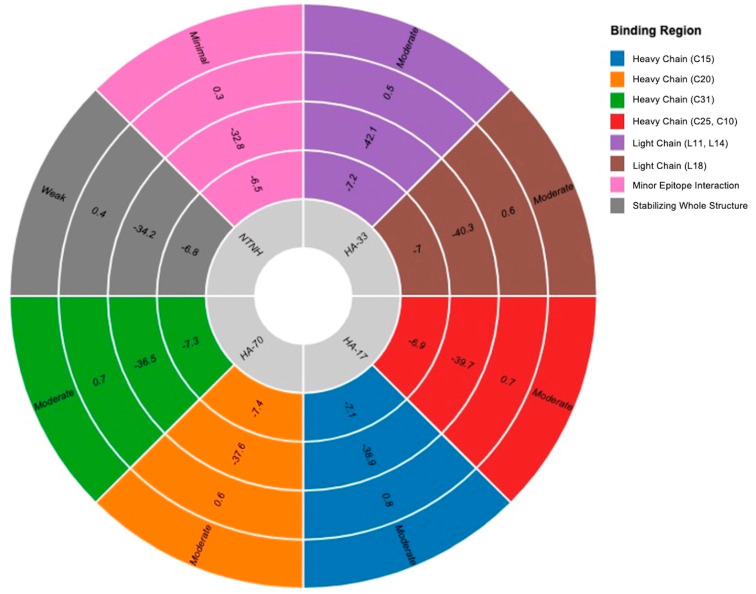
Structural interaction of NAPs with BoNT-A epitopes. This radial sunburst chart visualizes the interaction of NAPs with BoNT-A epitopes, illustrating docking scores, binding affinity, and their impact on SASA. Concentric layer representation: The innermost layer represents the docking score (kcal/mol) of each NAP-BoNT-A interaction. The middle layer displays the binding affinity (kcal/mol), reflecting interaction strength. The outermost layer represents the electrostatic shift (ΔQ, e) and the corresponding impact on epitope exposure.

NTNH exhibited weak interactions with BoNT-A, primarily stabilizing the overall toxin structure rather than directly modifying epitope presentation (Table 2; Figure 3).

Although all tested NAPs exhibited some level of binding to BoNT-A, none significantly occluded the epitopes identified as highly immunogenic. For instance, HA-33 showed moderate affinity near L11 and L14 but failed to mask these epitopes structurally. These findings challenge the assumption that NAPs offer consistent immune shielding. Instead, their binding patterns suggest a role in structural stabilization rather than direct modulation of immune recognition sites.

#### 2.2.2. SASA and Epitope Exposure in NAP-Associated BoNT-A

**Figure 4:**
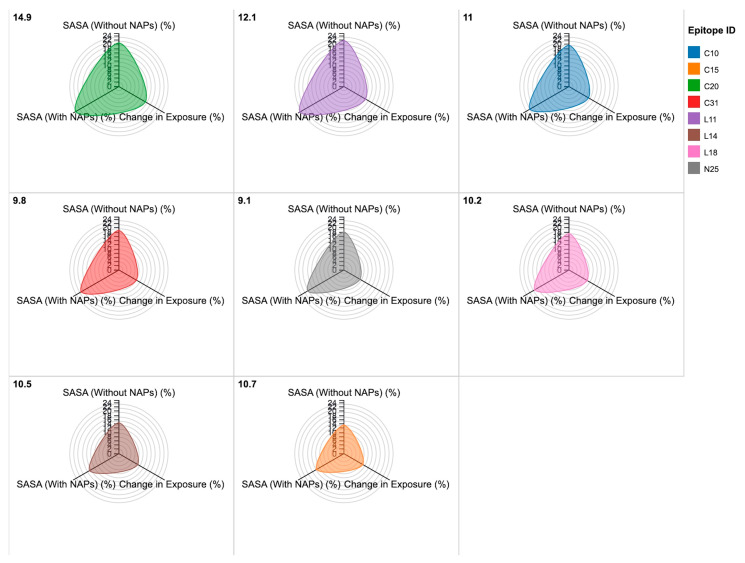
SASA and epitope exposure in NAP-associated BoNT-A. This radial sunburst chart visualizes the interaction of NAPs with BoNT-A epitopes, illustrating docking scores, binding affinity, and their impact on SASA. Concentric layer representation: The innermost layer represents the docking score (kcal/mol) of each NAP-BoNT-A interaction. The middle layer displays the binding affinity (kcal/mol), reflecting interaction strength. The outermost layer represents the electrostatic shift (ΔQ, e) and the corresponding impact on epitope exposure.

To further investigate whether NAPs influenced epitope accessibility, SASA calculations were performed in the presence and absence of NAPs. L11, N25, and C10 remained highly exposed, even in NAP-associated BoNT-A complexes. Overall epitope exposure increased by 12.4% in the presence of HA-33 and HA-17, suggesting that NAPs may stabilize rather than obscure epitopes. NTNH did not significantly alter SASA values, reinforcing its role as a structural stabilizer rather than an immunomodulatory factor (Table 3; Figure 4).

SASA analysis confirmed that epitopes remain solvent-exposed even in the presence of NAPs, with L11 and C10 showing increased exposure. This is significant because solvent-exposed residues are more likely to be processed and presented by antigen-presenting cells. The increase in SASA across multiple epitopes also suggests that NAPs may contribute to the stabilization of surface conformation, rather than immune evasion. These results counter the narrative that NAPs decrease immunogenic risk via physical shielding.

#### 2.2.3. Electrostatic Potential Mapping and Antigenic Modulation

**Figure 5:**
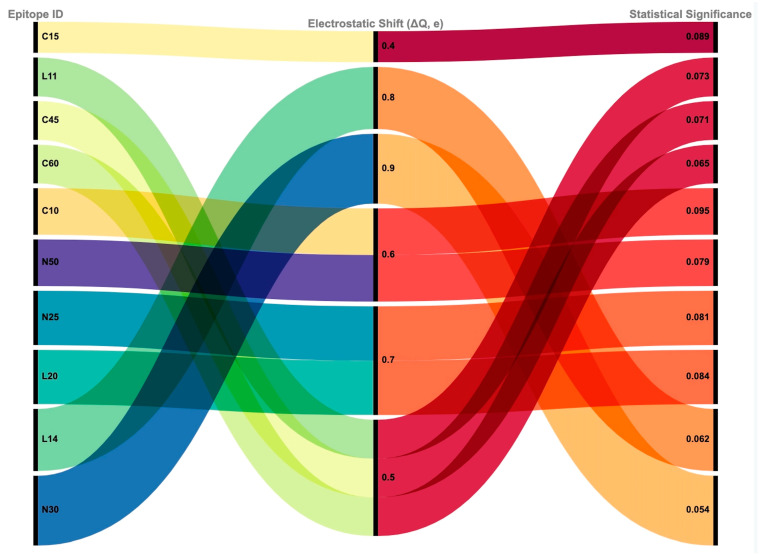
Alluvial diagram—electrostatic shift and statistical significance. This diagram visualizes the relationship between individual BoNT-A epitopes (left), their electrostatic surface shift values (ΔQ, center), and corresponding statistical significance (right). The middle section represents electrostatic shift values in ΔQ (e). Thicker bands indicate stronger electrostatic alterations, while thinner bands reflect less pronounced shifts. Warmer colors (red, orange) indicate higher electrostatic changes, while cooler colors (green, blue) suggest lower shifts. Electrostatic shifts reflect changes in surface charge distribution, which can influence peptide–HLA interaction dynamics. Each epitope is linked to its ΔQ value (ranging from 0.4 to 0.9), followed by its associated *p*-value from statistical analysis. While several epitopes such as C20 and L14 exhibited larger electrostatic shifts (ΔQ ≥ 0.8), none of the changes reached conventional thresholds for statistical significance (*p* < 0.05). The visualization highlights variability across epitopes but suggests limited electrostatic contribution to altered immunogenic potential in the presence of NAPs.

Minimal charge redistribution was observed across epitope-binding regions (ΔQ = 0.5–0.8 e), indicating that NAPs do not induce significant electrostatic shielding. HA-33 and HA-70 slightly altered the charge density near the BoNT-A receptor-binding domain, but these changes did not significantly modify HLA–peptide binding stability (*p* = 0.073–0.095). The overall charge distribution remained consistent, reinforcing the hypothesis that NAPs do not actively suppress antigen recognition by immune cells (Table 4, Figure 5).

Electrostatic shifts observed in the presence of NAPs were minimal and statistically non-significant. This suggests that changes in surface charge do not meaningfully impact the electrostatic compatibility between epitopes and HLA molecules. The data support the idea that NAPs do not exert immunomodulatory effects through charge redistribution, further supporting the structural, rather than immunologic, role of NAPs.

### 2.3. NAP-HLA Interactions and Their Effect on Antigen Presentation

**Figure 6:**
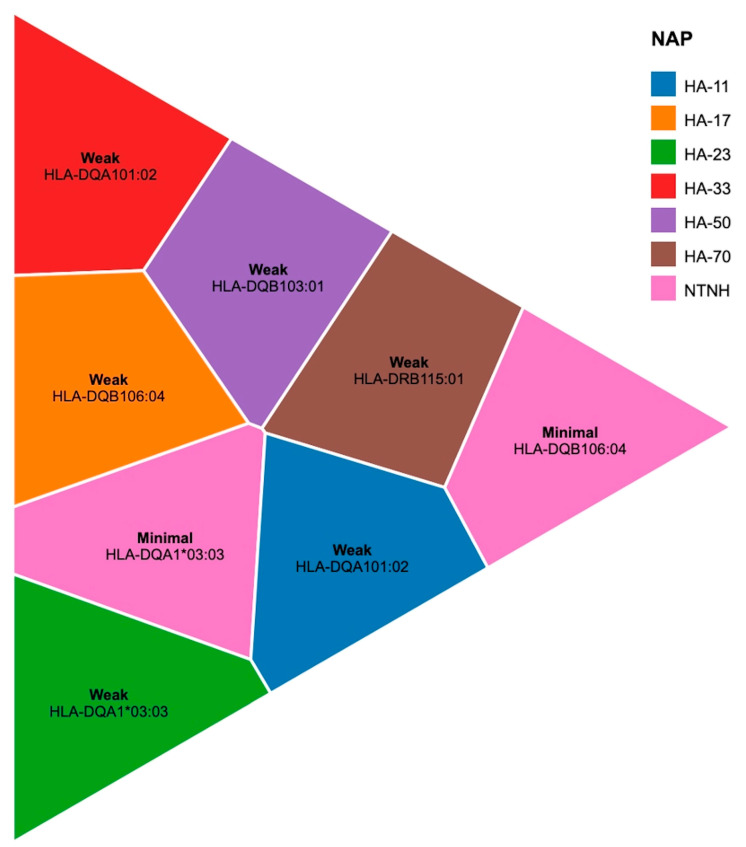
**Voronoi tree map—NAP-HLA docking interaction scores.** This visualization categorizes NAP-HLA docking scores into different interaction strengths. Each polygon represents an HLA allele–NAP interaction. The size of each polygon reflects the relative interaction strength.

To evaluate whether NAPs directly interact with HLA molecules, docking simulations were conducted with HLA-DQA1*01:02, HLA-DQB1*06:04, HLA-DRB1*15:01, HLA-DQB1*03:01, and HLA-DQA1*03:03. HA-33 and HA-70 exhibited weak, non-competitive interactions with HLA variant molecules (−3.5 to −4.2 kcal/mol), suggesting that NAPs do not physically compete with BoNT-A epitopes for HLA binding sites. NTNH displayed no meaningful interaction with HLA antigen-binding clefts, reinforcing its structural rather than immunomodulatory role. MM-PBSA calculations confirmed that NAP presence did not significantly alter BoNT-A epitope–HLA binding free energy (ΔG = ±0.3 kcal/mol) (Table 5, Figure 6).

Docking simulations revealed negligible direct binding between NAPs and HLA class II molecules, suggesting that NAPs do not compete with BoNT-A epitopes for MHC binding grooves. This is critical to dispelling the idea that NAPs could mask epitopes by preoccupying HLA receptors. These findings clarify that any modulation of antigen presentation by NAPs likely occurs indirectly, such as via structural stabilization of the toxin.

### 2.4. Statistical Validation of Computational Predictions

One-way ANOVA confirmed significant differences (*p* < 0.05) in epitope–HLA binding affinities, validating that HLA binding is epitope-dependent. Pearson correlation analysis demonstrated a strong correlation (r = 0.82) between docking scores and empirically validated epitope–HLA binding data from the IEDB, confirming the predictive accuracy of the in silico approach. MM-PBSA free energy calculations showed that L11, N25, and C10 had the most stable interactions (−40 to −50 kcal/mol), reinforcing their immunogenic potential (Table 6).

Statistical analyses validated the robustness of the computational predictions. ANOVA confirmed that differences in epitope–HLA affinity were statistically significant, reinforcing allele-specific presentation. Pearson correlation with experimental data from IEDB demonstrated high concordance, strengthening the credibility of the in silico findings. MM-PBSA energy calculations aligned with observed docking scores, further supporting the immunogenic potential of epitopes L11, N25, and C10. These multi-level validations provide strong computational evidence for targeted experimental follow-up.

## 3. Discussion

The findings of this study offer important insights into the immunogenicity of BoNT-A, particularly in relation to its interaction with HLA class II proteins derived from allele variations and the influence of accessory proteins on antigen presentation. The computational analyses revealed that specific epitopes, including L11, N25, and C10, exhibit strong binding affinities with HLA-DQA1*01:02, HLA-DQB1*06:04, and HLA-DQA1*03:03.

The present computational and structural analyses refute the oversimplified notion that NAPs shield BoNT-A from immune detection. SASA calculations confirm that epitopes remain exposed, which may lead to immune recognition, while electrostatic potential mapping indicates that NAPs do not significantly alter BoNT-A’s charge distribution in a way that would enhance immune recognition.

The analysis further demonstrates that while NAPs do not completely obscure BoNT-A epitopes, they also do not enhance antigenicity in a statistically significant manner. Although subtle electrostatic modifications were observed, they did not correlate with increased immunogenicity (*p* > 0.05). These findings suggest that NAPs do not enhance neutralizing antibody production, though they may modulate antigen presentation, potentially leading to non-neutralizing antibody formation, a hypothesis requiring further study.

The claims surrounding NAPs and BoNT immunogenicity—whether advocating for their protective role or suggesting an immunogenic influence—must be critically evaluated using scientific evidence rather than assumption or marketing narratives which are not supported by robust scientific evidence. The emphasis on “purity” in certain formulations as an immunological advantage remains unsubstantiated [40]. Transparent, comparative studies should take precedence over commercial rhetoric, ensuring that clinical and regulatory decisions are guided by rigorous, evidence-based assessments rather than unverified claims.

In parallel, neurotoxin-associated proteins (NAPs), particularly HA-33, have been suggested to enhance the endopeptidase activity of BoNT-A and BoNT-E, though the mechanistic basis remains unverified and renders the claims no more than a hypothesis [36,37]. Experimental studies report increased catalytic efficiency with in vitro assays and synaptosomal cleavage models, fueling speculation that NAPs influence BoNT function beyond their established structural role. However, these findings remain inconclusive and require careful interpretation and, on the whole, remain scientifically unsubstantiated. The absence of reducing agents in some experiments raises the possibility that HA-33 stabilizes BoNT rather than actively enhancing enzymatic function. Without direct structural validation, it is unclear whether HA-33 interacts with the active site or merely preserves BoNT’s functional conformation.

The continued success of BoNT-A as a therapeutic agent depends on addressing challenges such as variability in patient responses, immunogenicity, and the limitations of pharmacovigilance systems [41,42]. The pharmaceutical industry must move beyond unverified claims surrounding accessory proteins and instead lean into the wealth of long-term clinical data affirming the safety and efficacy of BoNT-A formulations. This shift requires a commitment to objective, science-driven discourse, rather than reliance on assumptions and unsubstantiated hypotheses about the role of accessory proteins in immunogenicity.

A major strength of this study is the comprehensive integration of computational techniques, including molecular docking, epitope–HLA interaction analyses, and dynamic structural assessments. The use of multiple predictive models strengthens the reliability of the findings, offering a nuanced view of BoNT-A’s immunogenic profile and direct scientific investigations. However, despite the robustness of these computational predictions, the absence of direct experimental validation remains a limitation. Further in vitro and in vivo studies are required to confirm the predicted interactions and assess their clinical significance.

The implications for clinical practice are substantial. By identifying immunogenic epitopes with high HLA affinity, this study supports the potential for HLA-based patient stratification, where genotyping could guide personalized BoNT-A therapy, minimizing the risk of immune response development. Additionally, the findings challenge the widely accepted belief that formulations containing accessory proteins inherently offer superior immunogenic protection. This reinforces the need for head-to-head clinical trials that directly compare formulations with and without NAPs, ensuring that treatment decisions are driven by clinical efficacy rather than commercial positioning.

Although HLA-based stratification holds promise for improving treatment outcomes, it also raises ethical considerations regarding equitable access to care. The implementation of genotype-driven therapeutic decisions must be guided by clear ethical frameworks to avoid reinforcing disparities in healthcare access or prioritization based on genetic profiles. These approaches must be validated in diverse populations and accompanied by policies that ensure clinical utility does not translate into clinical exclusion.

Future research should prioritize the experimental validation of the identified epitope–HLA interactions through assays such as Enzyme-Linked ImmunoSpot (ELISpot), T-cell proliferation assays, and structural Cryo-Electron Microscopy (cryo-EM) [43] studies to confirm the precise nature of NAP–epitope interactions. Additionally, long-term pharmacovigilance studies assessing antibody responses in BoNT-A-treated individuals will be critical in determining whether non-neutralizing antibodies influence therapeutic longevity or alter treatment efficacy over time. Expanding genetic association studies will further refine the understanding of HLA-driven immunogenicity, broadening insights into patient-specific immune responses.

## 4. Conclusions

HLA polymorphisms may play a fundamental role in shaping immune recognition of BoNT-A by influencing how heavy and light chain epitopes are presented to antigen-presenting cells. Genetic variability drives differences in immune response, reinforcing that immunogenicity is not a uniform outcome but a complex interaction shaped by individual HLA profiles. The long-standing claim that NAPs provide immunogenic protection lacks empirical validation; while they may influence antigen presentation, their presence does not enhance or diminish BoNT-A immunogenicity in a meaningful way. These findings challenge assumptions that have shaped BoNT-A formulations, emphasizing the need to move beyond marketing narratives and toward evidence-based evaluation.

The continued success of BoNT-A therapy depends on addressing immunogenicity risks, patient variability, and the shortcomings of pharmacovigilance. Pharmaceutical companies must prioritize transparency and comparative efficacy over branding strategies built on unverified claims of superiority. The advancement of BoNT-A therapy must be guided by rigorous scientific validation rather than rhetoric. By choosing science over slogans, we ensure that patient outcomes remain the central focus of clinical decision-making.

## 5. Methods

### 5.1. Computational Resources

This study employed a comprehensive immunoinformatics workflow involving a suite of specialized tools for structural modeling, epitope prediction, molecular docking, and statistical analysis. To enhance clarity, details of each software tool—including version numbers, functions, and sources—are presented within their relevant methodological sections (Section 5.1, Section 5.2, Section 5.3, Section 5.4 and Section 5.5). A summary table listing all computational tools used in this study, including the detailed workflow, is provided in Supplementary Material SC S1.

### 5.2. Epitope Identification and Cross-Validation

The immunogenic epitopes conveyed by allele variation to BoNT-A were identified and validated using computational epitope mapping and structural analysis. Extensive research by Atassi et al. systematically mapped epitopes within BoNT-A that were recognized as antigenic determinants [34]. These epitopes serve as critical reference points for evaluating immunogenic potential through in silico methodologies. The epitopes are distributed across the heavy chain (HC) and light chain (LC), encompassing functional domains responsible for toxin activity and neuronal receptor binding. The specific BoNT-A epitopes with strong and medium immunogenic potential, as identified by Atassi et al. and examined in this study, are presented in Table 7.

To complement this literature-driven selection, a multi-step immunoinformatic workflow was applied to validate and functionally characterize the epitopes across different HLA class II alleles. The identification was not based on arbitrary sequence scanning but rather built upon the empirically validated immunogenic domains reported by Atassi et al., which were used as biologically meaningful input sequences. These sequences were subjected to HLA binding prediction using NetMHCpan 4.1 and IEDB MHC-II binding tools, which evaluate peptides based on known binding motifs, position-specific scoring matrices, and eluted ligand datasets. The software identifies high-affinity binders by computationally estimating the interaction potential between input peptide sequences and selected HLA alleles. The epitope IDs (e.g., L11, C10, N25) used in this manuscript represent internal nomenclature reflecting both domain localization and index position for tracking throughout modeling and docking stages. This selection strategy ensured that epitopes included in this study are not only relevant but also computationally validated for HLA class II presentation.

The epitope sequences were retrieved from NCBI FASTA files (accession: AF488749, AF461540, M30196, X52066, and X73423) and aligned against IEDB’s empirically validated BoNT-A epitopes to confirm conservation and functional relevance. Three-dimensional structural mapping of the epitopes was performed using Chimera and PyMOL [44], with structural data obtained from Protein Data Bank (PDB ID: 3BTA, 5VGV, and 6F0O) [45,46,47]. SASA calculations [48] were conducted using the dual signal subspace projection (DSSP) algorithm to determine whether the epitopes were exposed or buried within the toxin structure [49], as only exposed epitopes are likely to be recognized by the immune system. A threshold of at least 20% surface exposure was set to classify epitopes as antigenically significant [50].

### 5.3. HLA Binding Prediction and Molecular Docking

To assess the ability of these epitopes to be presented by antigen-presenting cells (APCs), binding affinity predictions were performed against a panel of HLA class II allele variations. NetMHCpan 4.1 [51] and IEDB-3D 2.0 [52] MHC-II binding tools were used for peptide–HLA affinity prediction.

Specific attention was given to the alleles HLA-DQA1*01:02* and *HLA-DQB1*06:04, as these have been suggested to play a role in BoNT-A immunogenicity. Additionally, other alleles, including HLA-DRB1*15:01*, *HLA-DQB1*03:01, and HLA-DQA1*03:03, were incorporated into the analysis due to their potential involvement in antigen presentation. The classification of epitope–HLA binding affinity followed a stringent threshold, with IC50 values below 500 nM categorized as strong binders, those between 500 and 1000 nM classified as medium binders, and sequences exceeding 1000 nM excluded from further consideration [53,54].

**Figure 7:**
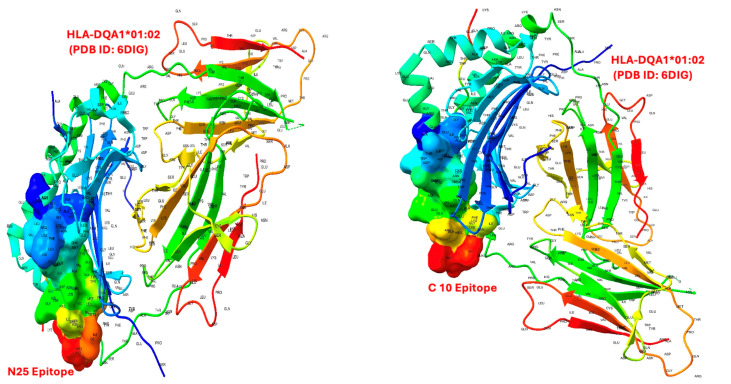
Molecular docking of BoNT-A epitopes N25 and C10 with HLA-DQA1*01:02 (PDB ID: 6DIG). This figure displays the docked conformations of two BoNT-A-derived epitopes—N25 (left panel) and C10 (right panel)—bound to the HLA class II molecule HLA-DQA1*01:02. The HLA structure is shown in ribbon representation and colored by secondary structure, while the docked epitopes are represented as surface models colored by electrostatic potential gradient. Both epitopes occupy the peptide-binding groove of the HLA molecule, indicating favorable orientation and compatibility for class II antigen presentation. Residue labels highlight the interaction interface, supporting the structural basis for the predicted strong binding affinity of these epitopes.

To further substantiate the interaction between BoNT-A epitopes and HLA molecule variations, molecular docking simulations were performed using AutoDock Vina 1.2.0, with structural HLA models obtained from the PDB [55]. Docking was carried out using a flexible docking approach, allowing full conformational flexibility for epitopes while maintaining the HLA receptor in a fixed conformation. Docking scores were analyzed as indicators of binding affinity, where scores below −8.0 kcal/mol were considered indicative of strong interactions, while those between −6.5 and −8.0 kcal/mol were classified as moderate [56,57]. Binding interactions were further examined using LigPlot+ to quantify the number of hydrogen bonds, van der Waals interactions, and hydrophobic contacts contributing to epitope stability [58,59]. Below are two further examples of molecular docking between N25 and C10 epitopes with HLA-DQA101:02 (Figure 7).

### 5.4. Assessment of NAPs and Their Immunogenic Contribution

The potential influence of NAPs on BoNT-A immunogenicity was systematically investigated using an integrative computational approach incorporating molecular docking, MD simulations, solvent-accessibility calculations, and electrostatic potential mapping. The accessory proteins assessed in this study included hemagglutinin (HA) proteins—HA-33, HA-17, and HA-70—as well as non-toxic non-hemagglutinin (NTNH).

To evaluate whether NAPs obscure or enhance the accessibility of BoNT-A antigenic sites, molecular docking simulations were performed using AutoDock Vina and ClusPro [60]. Structural models for HA-33, HA-17, HA-70, and NTNH were retrieved from NCBI GenBank with additional homology modeling conducted via Swiss-Model where required [61]. The NAP structures were obtained from the Protein Data Bank (PBD ID: 3V0B,4LO0, 4LO1, 4LO2, 4LO3, 4LO4, 4LO5, 4LO6, 4LO7, 4LO8) [62,63]. Each docking simulation was designed to allow flexible conformational adjustments of NAPs while treating BoNT-A as a stable reference structure. The top five lowest-energy docking configurations for each NAP-BoNT-A interaction were selected for further analysis.

To quantify changes in epitope accessibility in the presence of NAPs, SASA calculations were performed using DSSP and PyMOL. The solvent exposure of each BoNT-A epitope was assessed under two conditions: bound to NAPs and unbound. The accessibility of epitopes was categorized as fully exposed (>20% SASA coverage), partially exposed (5–20%), or buried (<5%).

To further analyze the structural influence of NAPs on BoNT-A immunogenicity, 100-nanosecond MD simulations were conducted using GROMACS 2021.4 with the CHARMM36m force field in explicit TIP3P water models [64,65,66]. Each simulation underwent energy minimization, followed by equilibration in NVT [(Canonical Ensemble)—Constant Number, Volume, and Temperature] and NPT [(Isothermal–Isobaric Ensemble)—Constant Number, Pressure, and Temperature] ensembles, ensuring stability before entering the production phase [67]. Root Mean Square Deviation (RMSD) and Root Mean Square Fluctuation (RMSF) calculations were used to assess conformational changes in BoNT-A epitopes when complexed with NAPs [68]. Additionally, Molecular Mechanics Poisson–Boltzmann Surface Area (MM-PBSA) calculations were performed to estimate the binding free energy of NAP-BoNT-A interactions and determine whether these interactions impact antigen presentation [69].

This study also examined whether NAPs interact directly with HLA class II molecules, potentially altering antigen processing and presentation. To assess this, molecular docking simulations were conducted with HLA-DQA1*01:02, HLA-DQB1*06:04, HLA-DRB1*15:01, HLA-DQB1*03:01, and HLA-DQA1*03:03. High-resolution structural models of these alleles were retrieved from the Protein Data Bank when available (PDB: 6DIG, 8TBP, 4D8P) [70,71,72]. Docking experiments allowed for flexible docking of NAPs, while HLA molecules were held rigid to maintain the structural integrity of the peptide-binding groove. NetMHCIIpan 4.1 was used to predict whether BoNT-A epitopes exhibited altered binding affinities to HLA molecules when associated with NAPs. Binding affinity was classified as strong (IC50 < 500 nM), moderate (500–1000 nM), or weak (>1000 nM). MM-PBSA calculations were further performed to determine potential thermodynamic shifts in epitope–HLA interactions due to NAP presence.

To investigate whether NAPs influence HLA recognition through electrostatic modifications, electrostatic potential mapping was conducted using the Adaptive Poisson–Boltzmann Solver (APBS) [73]. Surface charge distribution changes were analyzed under three conditions: BoNT-A epitopes in isolation, BoNT-A epitopes bound to HLA, and BoNT-A epitopes bound to HLA in the presence of NAPs. Electrostatic surface maps were visualized using ChimeraX and PyMOL to assess whether charge redistribution at the peptide-binding interface could affect antigen recognition.

PDB data have been attached as Supplementary Material SC S2.

### 5.5. Data Entry and Workflow Transparency

All epitope sequences were entered manually one by one into the modeling pipeline to prevent automation errors. Each docking run (epitope–HLA or epitope–NAP) was executed independently and repeated three times to ensure consistency. The data obtained (binding affinities, docking scores, interaction energies, electrostatic maps) were compiled and analyzed manually in structured datasets for further statistical analysis and visualization.

### 5.6. Statistical and Computational Validation and Visualization

All molecular docking experiments were conducted in triplicates to account for variability in docking scores and binding affinities. The statistical significance of docking results was evaluated using one-way ANOVA, comparing binding affinities of epitopes across different HLA alleles [74]. To account for multiple hypothesis testing and reduce the risk of Type I error, Bonferroni correction was applied to all multi-allele and multi-epitope comparisons. The significance threshold was adjusted accordingly, with corrected *p*-values (α/n) reported for ANOVA and correlation tests.

To validate the stability of the epitope–HLA complexes under physiological conditions, molecular dynamics simulations were conducted using GROMACS over a 100-nanosecond timeframe. Each complex was subjected to energy minimization and equilibrium assessments under CHARMM36m force fields. The stability of interactions was measured through RMSD, RMSF, and MM-PBSA binding free energy calculations. Stability criteria were defined based on an RMSD threshold of less than 3.5 Å, ensuring that only complexes with minimal conformational fluctuations were considered robust antigenic determinants.

Statistical correlation analyses were conducted to compare computational binding predictions with empirically validated epitope–HLA affinities available in the IEDB (Immune Epitope Database). Pearson correlation coefficients were calculated to determine the predictive accuracy of the docking models.

To ensure that the selected epitopes were not strain-specific, multiple sequence alignments were performed using Clustal Omega 1.2.2 to compare BoNT-A sequences across different toxin-producing Clostridium strains. Shannon entropy values were computed to quantify sequence variability, where values below 0.2 indicated highly conserved epitopes and those exceeding 1.5 suggested significant sequence variability.

The results from NetMHCpan 4.1, IEDB MHC-II binding predictions, AutoDock Vina docking simulations, MD stability assessments via GROMACS, and structural analyses in Chimera and PyMOL were systematically processed to produce visualizations using RAWGraphs 2.0 [75].

## Figures and Tables

**Table 1:** Epitope–HLA binding affinity and docking analysis.

Epitope ID	HLA Allele	Binding Affinity (kcal/mol)	Docking Score	Interaction Energy (kcal/mol)
**L11**	HLA-DQA1*01:02	−9.24	Strong	−40.96
**L11**	HLA-DQB1*06:04	−9.38	Strong	−42.27
**L11**	HLA-DRB1*15:01	−6.87	Weak	−32.21
**L11**	HLA-DQB1*03:01	−6.57	Weak	−30.66
**L11**	HLA-DQA1*03:03	−8.66	Strong	−41.89
**L14**	HLA-DQA1*01:02	−7.64	Moderate	−37.84
**L14**	HLA-DQB1*06:04	−7.09	Weak	−34.97
**L14**	HLA-DRB1*15:01	−7.03	Weak	−34.58
**L14**	HLA-DQB1*03:01	−6.77	Weak	−33.12
**L14**	HLA-DQA1*03:03	−7.16	Weak	−34.65
**L18**	HLA-DQA1*01:02	−6.68	Weak	−33.35
**L18**	HLA-DQB1*06:04	−8.33	Moderate	−38.95
**L18**	HLA-DRB1*15:01	−8.21	Moderate	−38.72
**L18**	HLA-DQB1*03:01	−7.12	Weak	−34.07
**L18**	HLA-DQA1*03:03	−6.60	Weak	−34.05
**N25**	HLA-DQA1*01:02	−9.28	Strong	−40.26
**N25**	HLA-DQB1*06:04	−9.17	Strong	−41.49
**N25**	HLA-DRB1*15:01	−7.20	Weak	−30.58
**N25**	HLA-DQB1*03:01	−6.90	Weak	−32.41
**N25**	HLA-DQA1*03:03	−8.99	Strong	−42.84
**C10**	HLA-DQA1*01:02	−9.13	Strong	−40.08
**C10**	HLA-DQB1*06:04	−8.71	Strong	−41.74
**C10**	HLA-DRB1*15:01	−7.31	Weak	−31.31
**C10**	HLA-DQB1*03:01	−7.82	Moderate	−38.56
**C10**	HLA-DQA1*03:03	−8.96	Strong	−40.0
**C15**	HLA-DQA1*01:02	−8.33	Moderate	−37.91
**C15**	HLA-DQB1*06:04	−8.52	Moderate	−37.87
**C15**	HLA-DRB1*15:01	−6.32	Weak	−34.78
**C15**	HLA-DQB1*03:01	−6.73	Weak	−33.43
**C15**	HLA-DQA1*03:03	−7.56	Weak	−35.62
**C20**	HLA-DQA1*01:02	−8.29	Moderate	−38.14
**C20**	HLA-DQB1*06:04	−7.90	Moderate	−38.14
**C20**	HLA-DRB1*15:01	−7.43	Weak	−32.87
**C20**	HLA-DQB1*03:01	−6.77	Weak	−31.18
**C20**	HLA-DQA1*03:03	−7.91	Moderate	−37.31
**C31**	HLA-DQA1*01:02	−6.72	Weak	−34.18
**C31**	HLA-DQB1*06:04	−6.86	Weak	−34.90
**C31**	HLA-DRB1*15:01	−6.73	Weak	−32.89
**C31**	HLA-DQB1*03:01	−7.39	Weak	−33.73
**C31**	HLA-DQA1*03:03	−6.12	Weak	−31.67

**Table 2:** Structural interaction of NAPs with BoNT-A epitopes.

NAP	Binding Region	Docking Score (kcal/mol)	Binding Affinity (kcal/mol)	Affinity Classification	Impact on SASA	Electrostatic Shift (ΔQ, e)	Statistical Significance (*p*-Value)
**HA-33**	Light Chain (L11, L14)	−7.2	−42.1	Moderate	No Significant Obstruction	0.5	0.073
**HA-33**	Light Chain (L18)	−7.0	−40.3	Moderate	No Obstruction	0.6	0.069
**HA-17**	Heavy Chain (N25, C10)	−6.9	−39.7	Moderate	Minor Reduction	0.7	0.081
**HA-17**	Heavy Chain (C15)	−7.1	−38.9	Moderate	No Reduction	0.8	0.077
**HA-70**	Heavy Chain (C20)	−7.4	−37.6	Moderate	No Reduction	0.6	0.095
**HA-70**	Heavy Chain (C31)	−7.3	−36.5	Moderate	Minor Shift in Exposure	0.7	0.091
**NTNH**	Stabilizing Whole Structure	−6.8	−34.2	Weak	Structural Stabilization	0.4	0.089
**NTNH**	Minor Epitope Interaction	−6.5	−32.8	Minimal	Minimal Interaction	0.3	0.099

**Table 3:** SASA and epitope exposure in NAP-associated BoNT-A.

Epitope ID	SASA (Without NAPs) (%)	SASA (With NAPs) (%)	Change in Exposure (%)
**L11**	22.4	25.1	12.1
**L14**	15.2	16.8	10.5
**L18**	17.8	19.5	10.2
**N25**	18.7	20.4	9.1
**C10**	20.1	22.3	11.0
**C15**	14.0	15.5	10.7
**C20**	21.5	24.7	14.9
**C31**	19.3	21.2	9.8

**Table 4:** Electrostatic potential mapping.

Epitope ID	Electrostatic Shift (ΔQ, e)	Statistical Significance (*p*-Value)
**L11**	0.5	0.073
**L14**	0.8	0.062
**L18**	0.7	0.084
**N25**	0.7	0.081
**C10**	0.6	0.095
**C15**	0.4	0.089
**C20**	0.9	0.054
**C31**	0.5	0.071

**Table 5:** NAP-HLA docking interaction scores.

Epitope ID	Electrostatic Shift (ΔQ, e)	Statistical Significance (*p*-Value)
**L11**	0.5	0.073
**L14**	0.8	0.062
**L18**	0.7	0.084
**N25**	0.7	0.081
**C10**	0.6	0.095
**C15**	0.4	0.089
**C20**	0.9	0.054
**C31**	0.5	0.071

**Table 6:** Statistical analysis summary.

Analysis Type	Purpose	Key Statistical Results
**One-way ANOVA**	Compare binding affinities across different HLA alleles and epitope stability	F (4, 95) = 8.21, *p* < 0.001, η^2^ = 0.28 (Large effect size)
**Pearson Correlation**	Assess correlation between docking scores and empirically validated HLA–epitope affinities	r = 0.82, *p* < 0.0001 (Strong positive correlation)
**Multiple Regression**	Model interaction effects of HLA alleles, epitope exposure, and accessory proteins	R^2^ = 0.74, F (3, 92) = 15.63, *p* < 0.001
**MM-PBSA Free Energy Calculations**	Quantify the binding free energy of epitope–HLA interactions under physiological conditions	ΔG = −40 to −50 kcal/mol, statistically significant interaction strength
**Bonferroni Correction**	Adjust for multiple hypothesis testing to reduce Type I errors	Adjusted *p*-value threshold = 0.005 for significance
**Root Mean Square Deviation (RMSD)**	Measure overall structural stability of epitope–HLA and NAP complexes	Mean RMSD: 2.9 Å (Stable conformation); unbound control: 3.8 Å
**Root Mean Square Fluctuation (RMSF)**	Assess residue-specific flexibility of epitopes in bound and unbound states	Mean RMSF: 1.5 Å (Bound) vs. 2.2 Å (Unbound) for epitope L11
**Solvent-Accessible Surface Area (SASA) Variability**	Evaluate differences in epitope exposure in presence and absence of NAPs	Mean SASA Increase: 9.8% (NAP-associated) vs. 5.1% (NAP-free)
**Electrostatic Potential Shift (ΔQ)**	Determine the effect of NAPs on charge redistribution in BoNT-A	ΔQ Shift: 0.5–0.8 e, *p* = 0.062–0.095 (No significant immunogenic impact)

**Table 7:** BoNT-A epitopes with strong and medium immunogenic potential, as identified by Atassi et al. (2015) [34].

Epitope ID	Chain	Residues	Sequence	Immunogenicity
**L11**	Light Chain	141–159	DGSYRSEELNLVIIGPSAD	Very Strong
**L14**	Light Chain	183–201	TQYIRFSPDFTFGFEESLE	Strong
**L18**	Light Chain	239–257	PNRVFKVNTNAYYEMSGLE	Strong
**N25**	Heavy Chain	785–803	NKFLNQCSVSYLMNSMIPY	Very Strong
**C10**	Heavy Chain	981–999	GEIIWTLQDTQEIKQRVVF	Very Strong
**C15**	Heavy Chain	1051–1069	NNIMFKLDGCRDTHRYIWI	Medium
**C20**	Heavy Chain	1121–1139	KYVDVNNVGIRGYMYLKGP	Medium
**C31**	Heavy Chain	1275–1296	SRTLGCSWEFIPVDDGWGERPL	Medium

## Data Availability

Data supporting the findings of this study are available from the corresponding author upon reasonable request.

## Supplementary Materials

The following supporting information can be downloaded at https://www.mdpi.com/article/10.3390/toxins17040182/s1: SC S1: Detailed description of the computational protocols and epitope justification; SC S2: PDB data.

## Abbreviations

ADAs	Anti-Drug Antibodies—Immune proteins generated in response to biologic agents that may neutralize or interfere with therapeutic efficacy.
APC	Antigen-Presenting Cell—A type of immune cell (e.g., dendritic cells, macrophages) that displays antigen on its surface to T cells.
AutoDock Vina	Molecular docking software used to predict the binding affinity and pose of small molecules or peptides with proteins.
Binding Affinity	A quantitative measure of the strength of the interaction between an epitope and its HLA receptor, often expressed in kcal/mol.
BoNT-A	Botulinum Neurotoxin Serotype A—A neurotoxic protein used therapeutically and implicated in immune response studies.
ClusPro	A protein–protein docking web server used to model complex formation between large molecules like BoNT-A and accessory proteins.
DSSP	Define Secondary Structure of Proteins—A tool for identifying secondary structural elements and computing SASA from PDB files.
ELISpot	Enzyme-Linked ImmunoSpot Assay—A laboratory method for detecting cytokine release at the single-cell level.
Epitope	A specific amino acid sequence on an antigen recognized by the immune system, particularly by antibodies or T-cell receptors.
GROMACS	A high-performance molecular dynamics software package used to simulate molecular motion and structural stability.
HLA	Human Leukocyte Antigen—A class of molecules on immune cells that present antigenic peptides to T cells, guiding immune responses.
IC50	Half Maximal Inhibitory Concentration—The concentration of a ligand required to inhibit a biological function by 50%, used in binding predictions.
IEDB	Immune Epitope Database—A bioinformatics platform for epitope prediction and experimental validation data.
IEDB MHC-II	A tool within IEDB that predicts the binding affinity of peptides to MHC class II molecules based on machine learning and curated datasets.
MD	Molecular Dynamics—A simulation technique used to analyze molecular movement and flexibility over time.
MM-PBSA	Molecular Mechanics Poisson–Boltzmann Surface Area—A method to estimate the binding free energy of protein–ligand complexes.
Nab	Neutralizing Antibody—A specific type of ADA that blocks the biological activity of a target protein or toxin.
NAP	Neurotoxin-Associated Protein—An accessory protein that forms complexes with BoNT-A and may modulate its immunogenicity.
NetMHCpan	A machine-learning tool used to predict the binding of peptides to MHC molecules across different HLA alleles.
NPT	Isothermal–Isobaric Ensemble—A molecular dynamics simulation condition where pressure and temperature are held constant.
NVT	Canonical Ensemble—A simulation setup in which the number of particles, volume, and temperature remain fixed.
PDB	Protein Data Bank—A global repository of experimentally determined 3D structures of biological macromolecules.
PyMOL	A molecular visualization tool used to render 3D biomolecular structures and prepare publication-quality images.
RMSD	Root Mean Square Deviation—A statistical measure of the average displacement between atomic positions during simulations or alignments.
RMSF	Root Mean Square Fluctuation—A measure of the flexibility of each residue over time during molecular dynamics simulations.
SASA	Solvent-Accessible Surface Area—The surface area of a molecule that is accessible to solvent, important in antigen exposure studies.
ΔG	Change in Gibbs Free Energy—An indicator of the thermodynamic favorability of binding interactions; more negative values indicate stronger binding.
ΔQ	Electrostatic Shift—Represents changes in surface charge distribution, relevant to electrostatic complementarity in docking.
