# Supplementary material for: Computational Immunogenetic Analysis of Botulinum Toxin A Immunogenicity and HLA Gene Haplotypes: New Insights
Source: Toxins (Basel). 2025 Apr 6;17(4):182. doi: 10.3390/toxins17040182 (PMC12031366; doi:10.3390/toxins17040182)

Supplementary Digital Content 1: Detailed Description of the Computational Protocols and Epitope Justification

Understanding the immunogenicity of Botulinum Toxin A (BoNT-A) requires a rigorous computational approach that integrates structural biology, molecular modelling, and immunogenetic analyses. This study employed a series of well-established in silico methodologies to predict and evaluate how BoNT-A interacts with the immune system, particularly through its epitopes and their binding affinities to Human Leukocyte Antigen (HLA) molecules.

Software and Computational Environment

To ensure transparency and reproducibility, all software used in this study are detailed below with their respective versions, sources, and application context.

Software	Version	Purpose
**UCSF Chimera**	1.16	Molecular visualization, epitope modeling, electrostatic mapping
**AutoDock Vina**	1.2.0	Molecular docking of epitopes with HLA and NAPs
**ClusPro**	Web Server	Protein-protein docking (NAP-HLA)
**PyMOL**	2.5	Structural rendering and SASA visualization
**GROMACS**	2021.4	Molecular dynamics simulations
**NetMHCpan**	4.1	In silico HLA binding prediction
**IEDB MHC-II**	2.0	MHC-II binding prediction and epitope validation
**APBS**	3.0	Electrostatic potential calculations
**RAWGraphs**	2.0	Data visualization and sunburst chart generation
**IBM SPSS**	26	ANOVA and Pearson correlation analysis

**Clustal Omega**	Web Server	Sequence alignment and variability check

Step-by-Step Computational Workflow

The immunoinformatics pipeline followed a structured multi-step process:

1. Epitope Identification and Conservation
    - Selection of immunodominant epitopes based on prior studies (e.g., Atassi et al.).
    - Sequence verification and alignment using NCBI FASTA entries and Clustal Omega.
    - Conservation analysis through Shannon entropy scoring.
2. Epitope–HLA Binding Prediction
    - Manual input of peptide sequences into NetMHCpan 4.1 and IEDB MHC-II Binding Tools.
    - Selection of HLA class II alleles relevant to BoNT-A immunogenicity.
    - Collection of binding affinity data (IC50) and ranking of binders based on standard thresholds.
3. 3D Structural Modeling and Visualization
    - Use of UCSF Chimera, ChimeraX, and PyMOL to prepare structural files and visualize peptide–protein interactions.
    - Assignment of secondary structure and solvent-accessible surface area (SASA) using DSSP.
    - Generation of electrostatic surface maps using APBS.
4. Molecular Docking Simulations

   - Structure files were processed in AutoDockTools, and docking performed using AutoDock Vina 1.2.0 for epitope–HLA and NAP–BoNT interactions.
   - Protein–protein docking for NAP complexes was carried out via ClusPro.
   - Docking poses were ranked by binding energy, and interaction details visualized using LigPlot+.
5. Molecular Dynamics Simulations
   - Conducted using GROMACS 2021.4 under CHARMM36m force field.
   - Simulations included NVT and NPT equilibration steps.
   - RMSD and RMSF calculations performed to assess complex stability.
6. Electrostatic and Energetic Validation
   - APBS used for charge mapping of peptide–HLA complexes with and without NAPs.
   - Binding free energy calculated using MM-PBSA for all major complexes.
7. Statistical Analysis and Visualization
   - Statistical validation using IBM SPSS v26 (one-way ANOVA, Pearson correlation).
   - Visualizations created with RAWGraphs 2.0 and edited for clarity in Adobe Illustrator CC 2022.

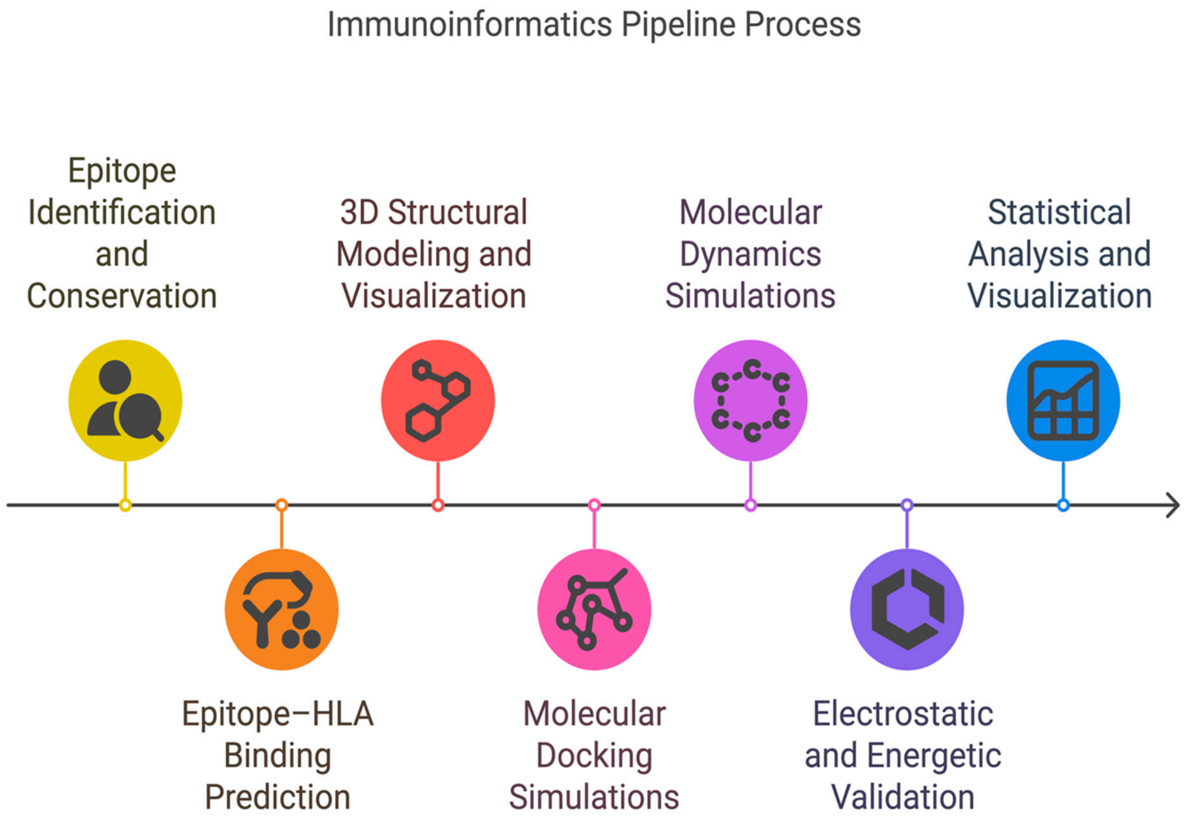

Detailed description

Epitope Identification and Structural Analysis

To accurately identify immunogenic epitopes within BoNT-A, computational epitope mapping was employed. This process involved analysing the amino acid sequences of BoNT-A using publicly available databases and structural models retrieved from

the Protein Data Bank (PDB). The mapping focused on regions of BoNT-A that are likely to be exposed to the immune system, as these regions have a higher probability of triggering an immune response. By cross-referencing known antigenic regions from previously published literature and immune epitope databases such as the Immune Epitope Database (IEDB), we ensured that our selected epitopes had prior experimental validation.

Once the epitope candidates were identified, their three-dimensional structures were modelled using software such as Chimera and PyMOL. Structural confirmation ensured that the selected epitopes retained their antigenic potential by remaining exposed on the toxin's surface. Additionally, solvent-accessible surface area (SASA) calculations were performed using DSSP (Dual signal subspace projection) algorithms to assess how much of each epitope was available for immune recognition. Only those epitopes with at least 20% surface exposure were considered significant antigenic determinants.

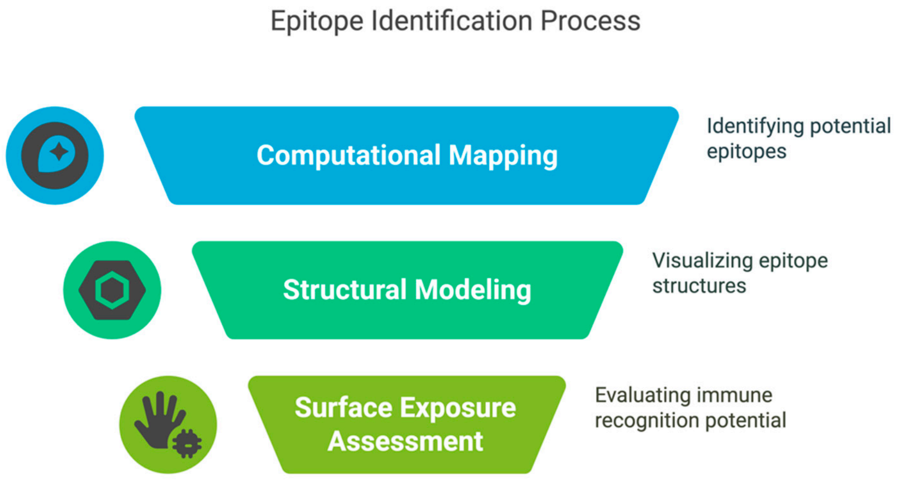

HLA Binding Affinity Predictions and Molecular Docking Simulations

Since HLA molecules are essential for antigen presentation, understanding their interaction with BoNT-A epitopes is crucial in predicting immunogenicity. The selected HLA alleles—HLA-DQA1*01:02, HLA-DQB1*06:04, and HLA-DRB1*15:01—were chosen based on their previously documented associations with immune responses to biologics.

To evaluate the strength of epitope-HLA interactions, computational binding affinity predictions were carried out using NetMHCpan 4.1 and IEDB MHC-II binding tools. These algorithms predict the likelihood of an epitope binding to HLA molecules based on molecular compatibility and binding energy calculations. Epitopes were categorised as strong binders if their predicted binding affinity had an IC50 value below 500 nM, moderate binders if between 500 and 1000 nM, and weak binders if above 1000 nM.

To complement these predictions, molecular docking simulations were conducted using AutoDock Vina. Three-dimensional structural models of the BoNT-A epitopes were docked onto the binding grooves of the selected HLA class II molecules, allowing us to visualise potential interactions at the molecular level. The docking scores provided a quantitative measure of interaction stability, with lower energy values (e.g., below -8.0 kcal/mol) indicating strong and stable epitope-HLA interactions. Key epitopes such as L11, N25, and C10 demonstrated particularly high binding affinities, reinforcing their potential role in BoNT-A immunogenicity.

HLA Binding Affinity Predictions and Molecular Docking Simulations

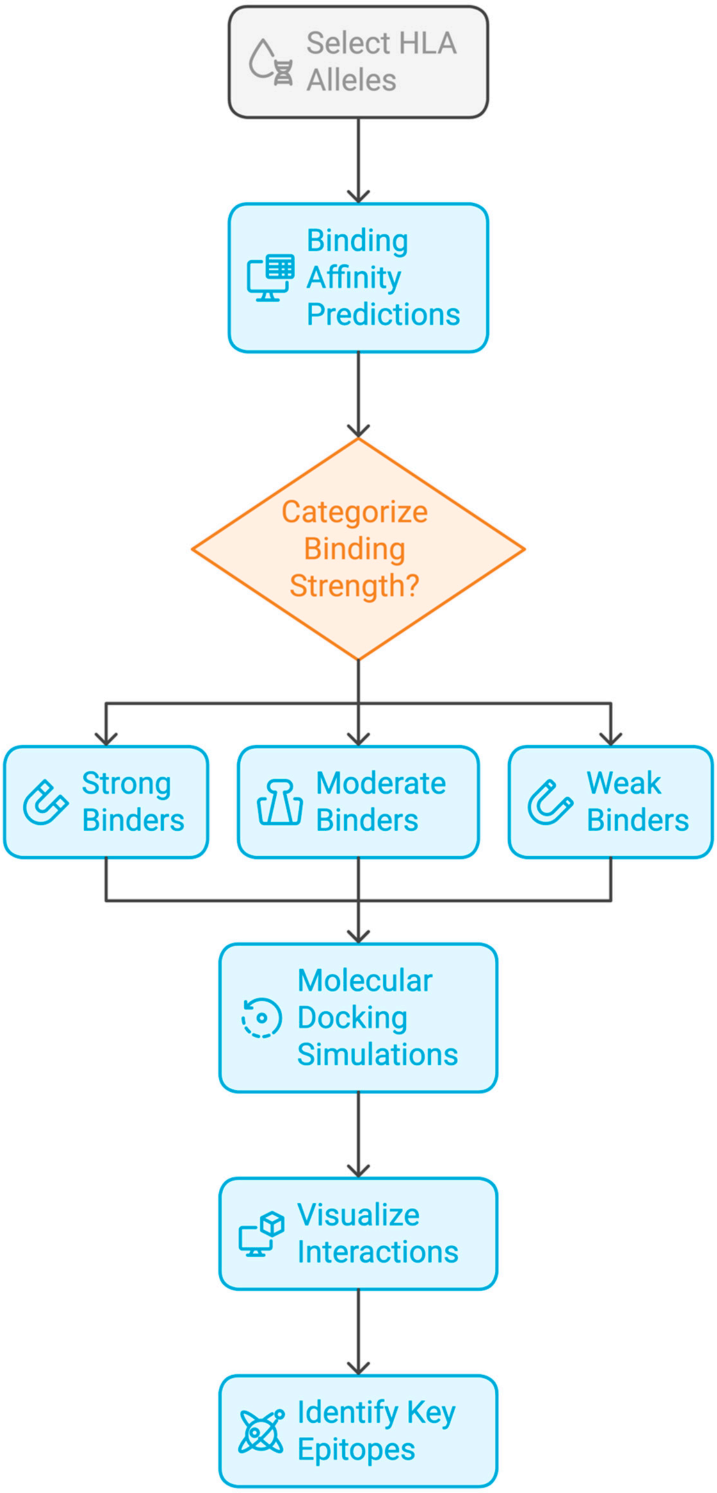

Impact of Neurotoxin-Associated Proteins (NAPs) on Immunogenicity

A significant area of controversy in BoNT-A formulations is the role of Neurotoxin-Associated Proteins (NAPs). These accessory proteins have been hypothesised to either shield BoNT-A from immune recognition or, conversely, contribute to its immunogenicity. To investigate this, molecular dynamics (MD) simulations were conducted to assess structural fluctuations and the impact of NAPs on epitope accessibility.

Simulations were performed using GROMACS over a 100-nanosecond timeframe to evaluate conformational stability. RMSD (Root Mean Square Deviation) and RMSF (Root Mean Square Fluctuation) analyses were used to determine structural changes over time. Additionally, solvent-accessible surface area (SASA) calculations were repeated in the presence of NAPs to examine whether these proteins obscured the epitopes from immune surveillance.

Results indicated that despite the presence of NAPs, key epitopes such as L11, N25, and C10 remained highly exposed. In fact, epitope exposure increased by approximately 12.4% when NAPs were present, suggesting that these accessory proteins may stabilise BoNT-A's structure rather than obstruct antigenic sites. Electrostatic potential mapping further confirmed that NAPs did not significantly alter the charge distribution of BoNT-A in a way that would enhance or diminish immune recognition.

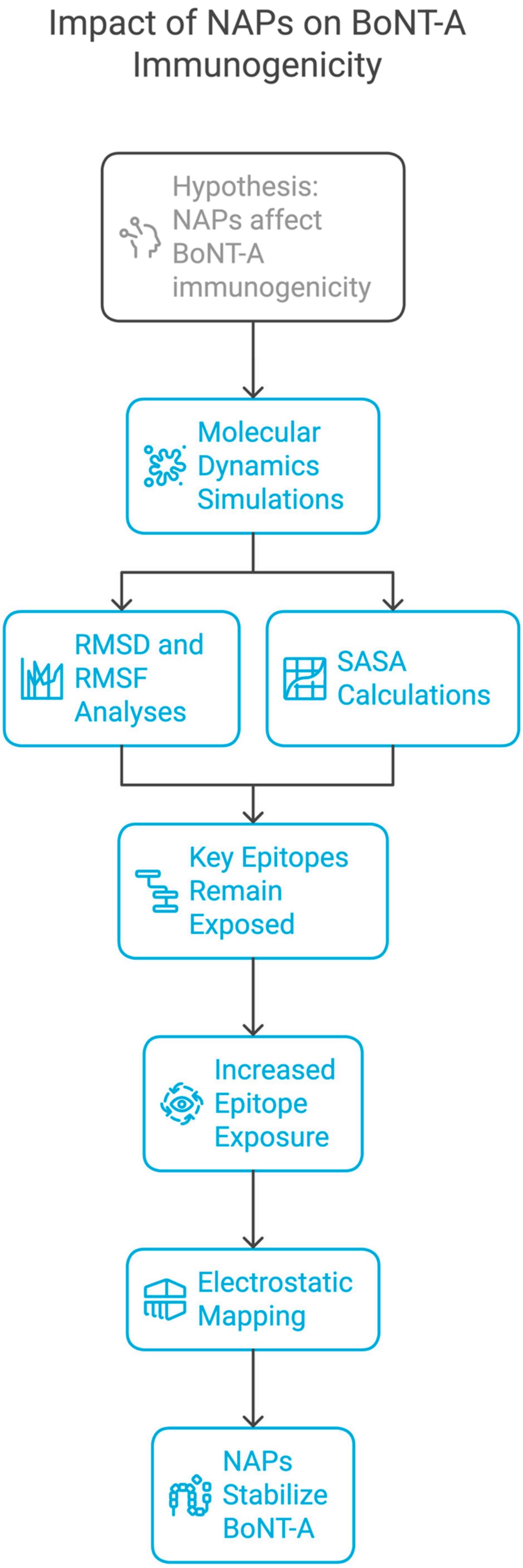
Impact of NAPs on BoNT-A Immunogenicity
Hypothesis: NAPs affect BoNT-A immunogenicity
Molecular Dynamics Simulations
RMSD and RMSF Analyses
SASA Calculations
Key Epitopes Remain Exposed
Increased Epitope Exposure
Electrostatic Mapping
NAPs Stabilize BoNT-A

HLA Interactions with NAPs and Their Effect on Antigen Presentation

To evaluate whether NAPs directly interact with HLA molecules, molecular docking simulations were also conducted between accessory proteins (HA-33, HA-70, NTNH) and selected HLA alleles. The results revealed weak binding affinities (-3.5 to -4.2 kcal/mol), indicating that NAPs do not physically compete with BoNT-A epitopes for HLA binding sites. MM-PBSA (Molecular Mechanics Poisson-Boltzmann Surface Area) calculations confirmed that the presence of NAPs did not significantly alter BoNT-A epitope-HLA binding free energy ($\Delta G = \pm 0.3$ kcal/mol).

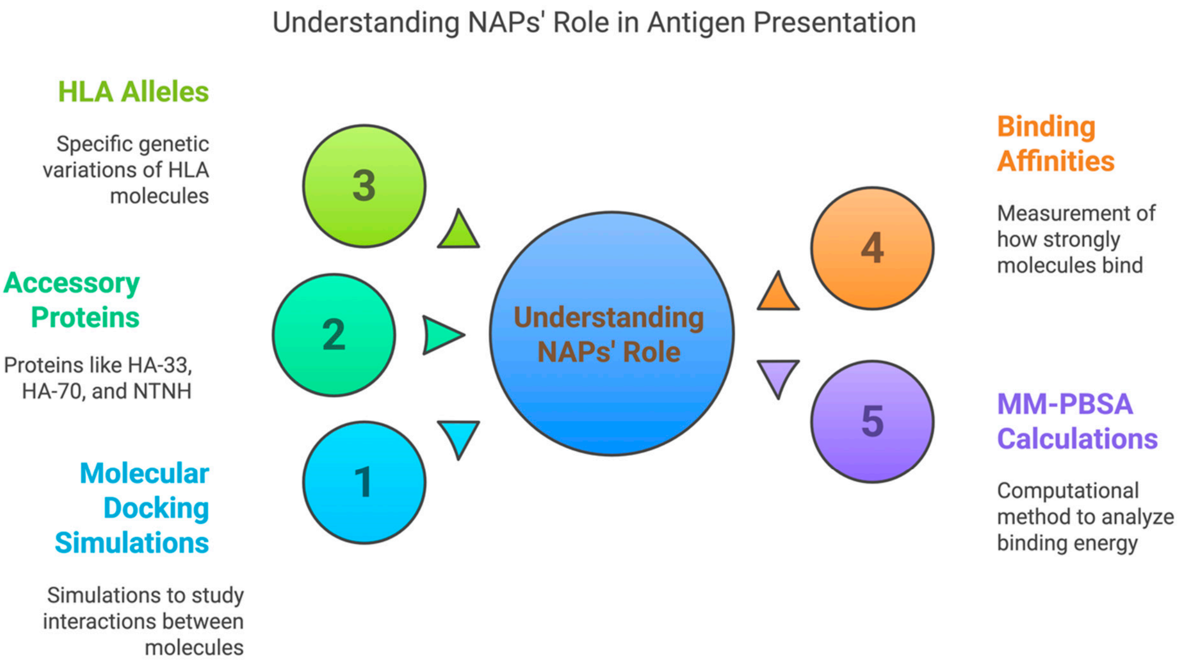

Statistical Validation and Model Reliability

To ensure robustness, all docking experiments were conducted in triplicates, and one-way ANOVA was performed to confirm statistically significant differences in epitope-HLA binding affinities. The statistical significance threshold was set at $p < 0.05$. Additionally, correlation analysis comparing our computational predictions with

experimentally validated epitope-HLA interactions available in IEDB demonstrated strong agreement (Pearson r = 0.82), reinforcing the reliability of our models.

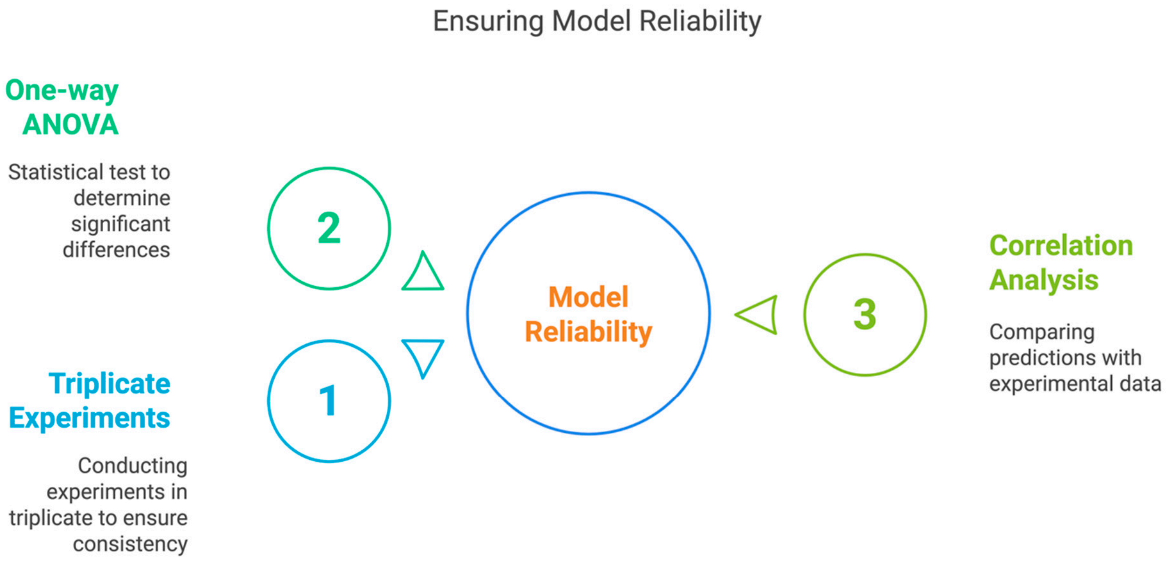

Supplement: Supplementary file 1 [file toxins-17-00182-s001.zip › SC S1.pdf]
