# Supplementary material for: Computational Immunogenetic Analysis of Botulinum Toxin A Immunogenicity and HLA Gene Haplotypes: New Insights
Source: Toxins (Basel). 2025 Apr 6;17(4):182. doi: 10.3390/toxins17040182 (PMC12031366; doi:10.3390/toxins17040182)
Supplement: Supplementary file 1 [file toxins-17-00182-s001.zip › SC S2.pptx]

## Slide 1
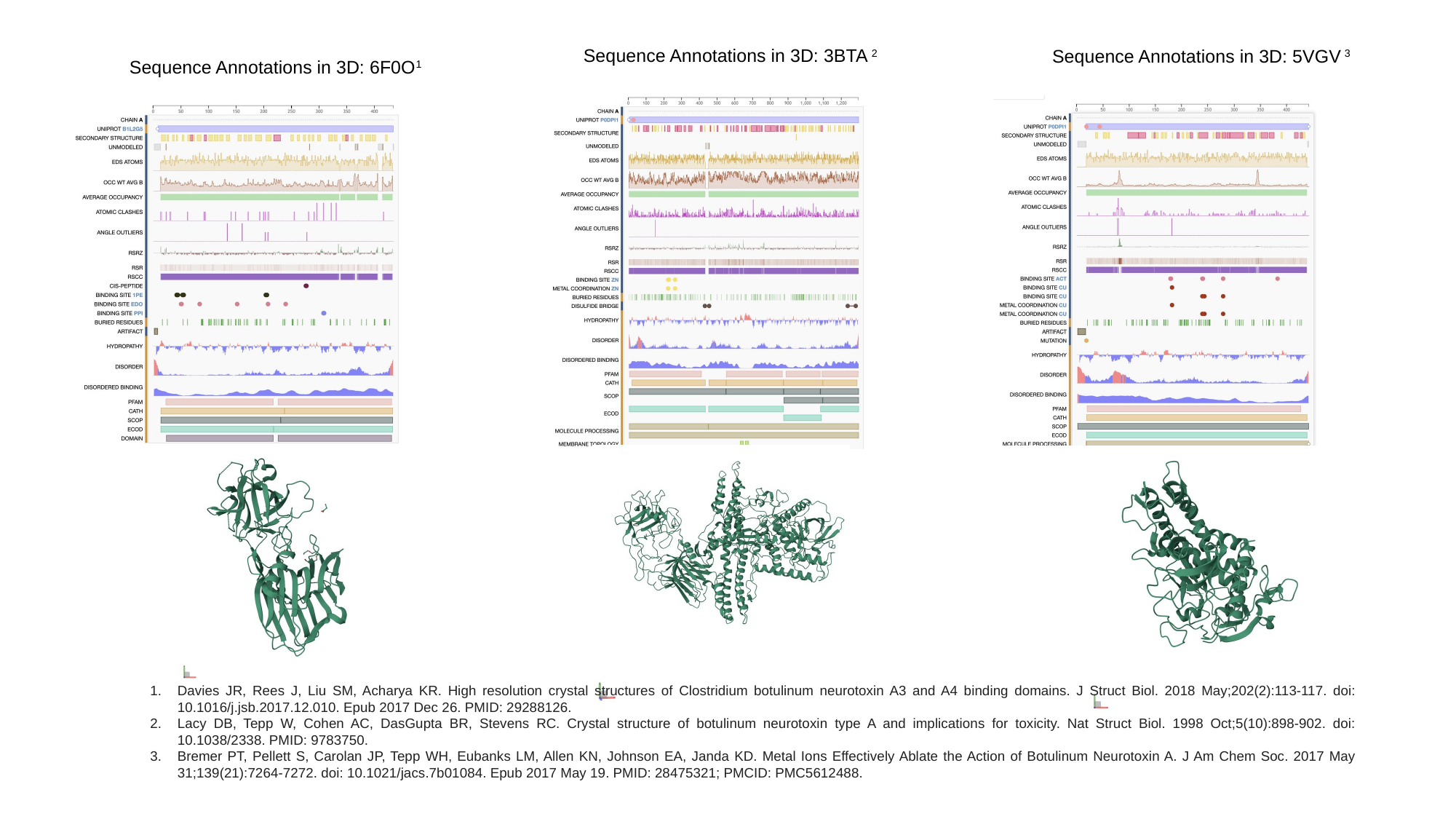

Sequence Annotations in 3D: 3BTA 2
Sequence Annotations in 3D: 5VGV 3
Sequence Annotations in 3D: 6F0O1
Davies JR, Rees J, Liu SM, Acharya KR. High resolution crystal structures of Clostridium botulinum neurotoxin A3 and A4 binding domains. J Struct Biol. 2018 May;202(2):113-117. doi: 10.1016/j.jsb.2017.12.010. Epub 2017 Dec 26. PMID: 29288126.
Lacy DB, Tepp W, Cohen AC, DasGupta BR, Stevens RC. Crystal structure of botulinum neurotoxin type A and implications for toxicity. Nat Struct Biol. 1998 Oct;5(10):898-902. doi: 10.1038/2338. PMID: 9783750.
Bremer PT, Pellett S, Carolan JP, Tepp WH, Eubanks LM, Allen KN, Johnson EA, Janda KD. Metal Ions Effectively Ablate the Action of Botulinum Neurotoxin A. J Am Chem Soc. 2017 May 31;139(21):7264-7272. doi: 10.1021/jacs.7b01084. Epub 2017 May 19. PMID: 28475321; PMCID: PMC5612488.

## Slide 2
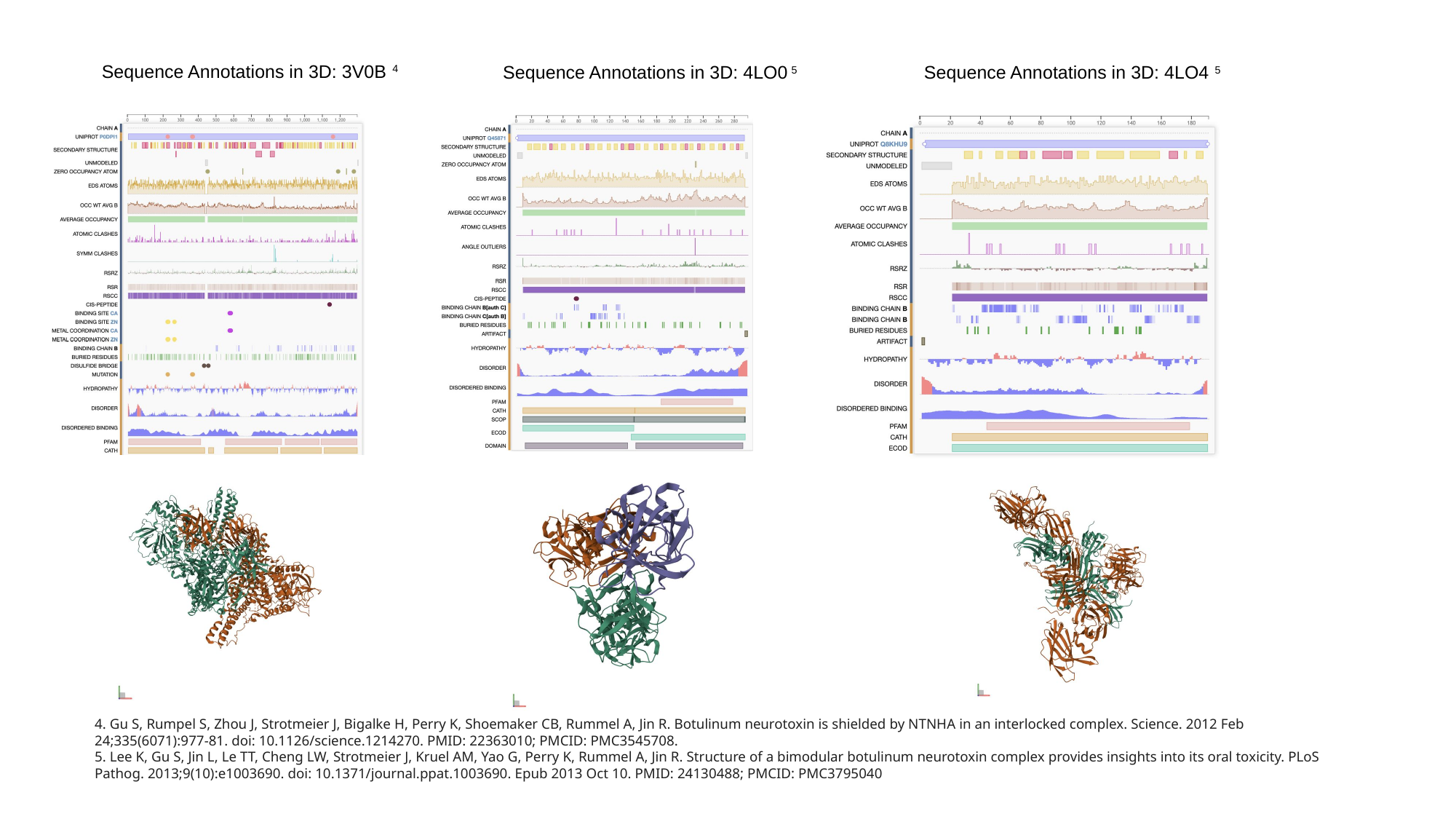

Sequence Annotations in 3D: 3V0B 4
Sequence Annotations in 3D: 4LO0 5
Sequence Annotations in 3D: 4LO4 5
4. Gu S, Rumpel S, Zhou J, Strotmeier J, Bigalke H, Perry K, Shoemaker CB, Rummel A, Jin R. Botulinum neurotoxin is shielded by NTNHA in an interlocked complex. Science. 2012 Feb 24;335(6071):977-81. doi: 10.1126/science.1214270. PMID: 22363010; PMCID: PMC3545708.
5. Lee K, Gu S, Jin L, Le TT, Cheng LW, Strotmeier J, Kruel AM, Yao G, Perry K, Rummel A, Jin R. Structure of a bimodular botulinum neurotoxin complex provides insights into its oral toxicity. PLoS Pathog. 2013;9(10):e1003690. doi: 10.1371/journal.ppat.1003690. Epub 2013 Oct 10. PMID: 24130488; PMCID: PMC3795040
